# Low serum vitamin D and early dental implant failure: Is there a connection? A retrospective clinical study on 1740 implants placed in 885 patients

**DOI:** 10.15171/joddd.2018.027

**Published:** 2018-09-18

**Authors:** Francesco Guido Mangano, Sina Ghertasi Oskouei, Ana Paz, Natale Mangano, Carlo Mangano

**Affiliations:** ^1^Department of Medicine and Surgery, Dental School, University of Varese, Italy; ^2^Faculty of Dentistry, Tabriz University of Medical Sciences, Tabriz, Iran; ^3^Private Practice, Lisbon, Portugal; ^4^Division of Endocrinology and Metabolism, Moriggia Pelascini Hospital, Gravedona ed Uniti, Italy,; ^5^Department of Dental Sciences, University Vita Salute San Raffaele, Milan, Italy

**Keywords:** Vitamin D, failures, implants, infections, osseointegration

## Abstract

***Background.*** Since osseointegration depends on bone metabolism, low levels of vitamin D in the blood may negatively affect bone formation around dental implants. To date, only a few studies have investigated the possible connection between serum levels of vitamin D and early dental implant failure (EDIF), i.e. failure that occurs within 4 months after placement, before the connection of the prosthetic abutment. The aim of this study was to investigate whether there is a relationship between low serum levels of vitamin D and EDIF.

***Methods.*** Data used for this retrospective study were derived from the records of a private dental clinic. Inclusion criteria were patients who had been treated with dental implants, inserted with a submerged technique from January 2003 to December 2017. EDIF was the outcome of this study. Chi-squared test was used to investigate the effect of patient-related variables (age, gender, smoking habit, history of periodontal disease and serum levels of vitamin D) on EDIF.

***Results.*** Originally, 885 patients treated with 1,740 fixtures were enrolled in this study. Overall, 35 EDIFs (3.9%) were reported. No correlation was found between EDIF and the patients' gender (P=0.998), age (P=0.832), smoking habit (P=0.473) or history of periodontal disease (P=0.386). Three EDIFs (11.1%) were reported in 27 patients with serum levels of vitamin D <10 ng/mL, 20 EDIFs (4.4%) in 448 patients with levels between 10 and 30 ng/mL, and 12 EDIFs (2.9%) in 410 patients with levels >30 ng/mL. Although there was a clear trend toward an increased incidence of EDIF with lowering of serum vitamin D levels, no statistically significant difference (P=0.105) was found among these three groups.

***Conclusion.*** Within its limitations (retrospective design, low number of patients with severe blood levels of vitamin D enrolled), this study failed to demonstrate a significant relationship between low serum levels of vitamin D and increased risk of EDIF. However, since a dramatic increase in EDIFs with lowering of vitamin D levels in the blood has been reported, further clinical studies with appropriate design (prospective or randomized controlled studies on a larger sample of severely deficient patients) are needed to better investigate this topic

## Introduction

Dental implants are today considered a successful treatment for restoring function and aesthetics.^1,2^ In fact, dental implant treatment has proven to be a predictable modality for replacing missing and failing teeth with various types of fixed and removable dental prostheses,^3,4^ with high survival rates even in the long term.^4,5^

Osseointegration, i.e. the formation of a direct interface between implant and bone, is key for the success of a dental implant.^5^ It is important that the implant be integrated into the bone during the initial healing period; this results in a clinically asymptomatic fixation under functional load, and this integration has to be maintained over time.^1,6^

Osseointegration of dental implants depends on several different factors: surgical and prosthetic factors (surgical technique^5,7^ and operator experience, timing and type of prosthetic loading,^8^ and quality of prosthetic rehabilitation^4^), implant-related factors (material, design,^9^ and surface^10^), and patient-related factors (bone volume/quality at the recipient site and host response^5,6,11^).

In recent years, research has focused mainly on surgical and prosthetic protocols, and on the characteristics of the implant, in order to further reduce implant failure rates.^9,10^ Therefore, new implant design and threads have been evaluated, in an effort to improve implant stabilization and to reduce the incidence of failures.^11^ In addition, a series of implant modifications have recently been introduced to accelerate and enhance the process of osseointegration, such as micro- and nano-topographical (surface) treatments, and addition of growth (and other) factors; all these modifications can help to improve osteogenic differentiation of bone marrow stromal cells (BMSCs)^12^ and to stimulate osteoblast activity.^13^

Despite the fact that all these improvements have contributed to the increase in the survival rate of dental implants, and therefore to make possible the application of surgical and prosthetic protocols that were previously considered high-risk, such as the immediate placement of implants in fresh post-extraction sockets^2,11,14^ and the immediate functional loading,^15,16^ unfortunately there is a percentage of implant failures which appear to be difficult to resolve.^17^

Based on chronological criteria, failures can be classified as either “early dental implant failures” (EDIFs) or “late dental implant failures” (LDIFs). EDIFs are due to unsuccessful osseointegration, indicating impaired bone healing, whereas LDIFs are due to loss of osseointegration. EDIFs and LDIFs appear to have different etiologies.^17,18^ LDIF is generally associated with infection (peri-implantitis), occlusal overloading, and failure or fracture of the implant components.^19,20^ EDIF is thought to usually be caused by factors such as inappropriate surgical and prosthetic protocols, surgical complications, low bone volume or quality at the recipient site, or habits (smoking and parafunctions) that, along with systemic conditions, can jeopardize osseointegration.^21,22^ In particular, early failures due to lack of osseointegration are among the worst problems in modern implantology. They appear to be difficult to resolve and, since they seem to occur in particular categories of patients, they might be related to the patient's systemic health condition.^17,21,22^ Recognition of systemic risk factors may reduce the failure rate and increase the predictability of dental implant treatment.^17,18,21,22^ Some factors in particular, such as vitamin D deficiency in the blood, could play an important role in the occurrence of EDIFs, but unfortunately are neglected by the dental literature.^23^

Vitamin D in its inactive form (vitamin D3 or cholecalciferol) is a steroid hormone that is acquired via diet or synthesized in the skin from cholesterol with adequate exposure to the sun (ultraviolet light).^24^ Cholesterol is converted to pre-vitamin D3 and then isomerized to vitamin D3. After binding to vitamin D-binding carrier protein, vitamin D3 is transported to the liver, where it is enzymatically hydroxylated by CYP27A1, generating 25-hydroxy vitamin D3 (calcidiol, or 25OHD3).^24^

Vitamin D acts as a hormone and is vital for the health of the brain,^25^ for the cardiovascular and respiratory tract,^26,27^ the skin, and the immune and endocrine systems.^28^ It is well known that vitamin D deficiency can impair the correct immune response to oral microbial infections, increasing the risk of periodontitis.^29^ Moreover, vitamin D plays an important role in the metabolism of bone.^30^ In the bone, vitamin D stimulates the activity of osteoclasts and increases the production of extracellular matrix proteins by osteoblasts.^30^

Today, vitamin D is considered deficient when serum 25(OH) levels are <10 ng/mL, insufficient when serum levels are 10–30 ng/mL, and optimal with serum levels of >30 ng/mL.^31^ Vitamin D deficiency, which can result from inadequate dietary intake together with insufficient exposure to sunlight, is today a worldwide public health concern.^31-33^ In fact, vitamin D deficiency is prevalent in the general population, particularly in the north of Europe;^32,33^ and in the north of Italy up to 80% of the population can be deficient during the winter season when exposure to the sun is lower.^31^ This deficiency increases with age and encompasses the vast majority of the elderly population of northern Italy who are not taking any supplements.^31,34^

As osseointegration of dental implants also depends on bone metabolism, there is the possibility that low levels of vitamin D in the blood can negatively affect healing processes and new bone formation on the implant surface.^35,36^

However, to date, there are only a few studies in specialized dental journals that investigate the correlation between vitamin D levels and EDIF.^37-48^ Most of the studies were have been carried out on animal models,^37-46^ with only a few on humans.^45-48^

In a recent retrospective clinical study, we tried to investigate whether there is a correlation between EDIF and low serum levels of vitamin D.^48^ Although our study failed to prove an effective link between low serum levels of vitamin D and an increased risk of EDIF, it did show a tendency to a higher incidence of early failures in subjects with vitamin D deficient states.^48^ We therefore decided to continue our retrospective study, including patients treated during the year 2017, to gather more data and to further investigate any possible link between low blood levels of vitamin D and EDIF.

## Methods

### Data collection

All the patients who had been treated with Morse taper connection dental implants (Leone Implant System^®^, Florence, Italy) at one particular dental center (Gravedona, Como, Italy), from January 2003 through December 2017, were screened for enrolment in the present retrospective study, as previously reported.^48^ The inclusion criteria were: patients >18 years of age at surgery, with good general oral hygiene, and without any bone regenerative therapy before implant placement. Patients with insufficient oral hygiene, uncontrolled diabetes mellitus, immunodeficient states, bleeding disorders, alcohol and/or drug abuse, or those who underwent radiotherapy and chemotherapy treatments, and those who were pregnant or had incomplete medical records were excluded from this study. The data used for the present clinical study were retrieved from the medical records of patients that met the aforementioned inclusion criteria and that did not present any of the aforementioned exclusion criteria. The medical records included information such as gender, age at time of surgery, smoking habits, history of chronic periodontal disease, and serum vitamin D levels. In fact, blood tests were requested two weeks prior to surgery to measure vitamin D levels of all the implant patients. The medical records also included information regarding the implant site (maxilla or mandible) and location (incisor, canine, premolar and molar), diameter and length of the fixture, prosthesis type, and loading conditions. Finally, any implant failure was also registered in the records, including the cause (lack of osseointegration in the absence of infection, implant failure due to progressive bone loss caused by prosthetic overload and infection of the peri-implant tissues or peri-implantitis). The failures were classified as either “early dental implant failures” (EDIFs) or “late dental implant failures” (LDIFs). EDIFs were failures occurring in the early healing period, within 4 months after implant placement and before the placement of restoration. LDIFs were late failures that occurred after loading. The present retrospective study was conducted following the principles highlighted in the Helsinki Declaration on research on human subjects (2003) and approved by the Ethics Committee of the University of Insubria (2013) under the code 0034086.

### Implants insertion protocol

All the implants were placed under the same strict protocol by the same clinician, who had more than 25 years’ experience in implant dentistry. The implants were placed after raising a full-thickness mucoperiosteal flap. The implant site preparation and implant placement were performed according to the manufacturer’s instructions and in compliance with modern surgical protocols. Cover screw was placed after placement and all the implants were submerged. The patients were prescribed antibiotics after surgery: 2 g of amoxicillin (or 600 mg of clindamycin in patients allergic to penicillins) for a 6-day period. Postoperative pain was controlled with nimesulide 100 mg daily for 2 days. Detailed instructions on oral hygiene were given to the patients, and 0.12% chlorhexidine mouth rinse was prescribed twice a day for 6 days. Ten days after surgery, the patients were recalled for suture removal. Then, for a period of 4 months, the implants were left to heal submerged, to allow optimal healing and achieve osseointegration. After 4 months, the patients were recalled for the implant to be uncovered. The healing abutment replaced the cover screws, and sutures were placed. Two weeks later, the sutures were removed and impressions were taken for the manufacture of temporary restorations. Temporary restorations were maintained for a period of 3 months, in order to monitor the response of the implant. The peri-implant tissues and masticatory load were also monitored. Once the 3-month period had passed, temporary restorations were replaced with final restorations made of metal porcelain and cemented with a zinc oxide–eugenol cement. All the patients were included in a follow-up protocol, with at least two annual check-ups.

### Outcome of the study

The outcome studied was EDIF, which occurred within 4 months after implant placement and, therefore, before the placement of the prosthetic restoration and the functionalization of the fixture. EDIFs were classified into two different categories: (a) early failures due to lack of osseointegration and subsequent implant mobility, in the absence of clinical signs of infection; and (b) early failures due to infection of the bone tissue around the implant, with inflammation of peri-implant tissues (peri-implantitis) and the presence of pain, swelling, fistula, pus, and/or exudate, pocket depth >6 mm with bleeding, and marginal bone resorption >2.5 mm.

### Statistical analysis

Data were recorded on a generic spreadsheet (Excel^®^, Microsoft, Redmond, MA, USA) which was used for the descriptive, quantitative and qualitative analyses. The means, standard deviations, medians and confidence intervals were calculated for the quantitative variables (e.g. patient’s age and vitamin D levels in serum). To calculate implant survival, a patient-based technique was used. The “event” in this analysis was early implant failure; thus, if a patient received more than one implant, the occurrence of even a single implant failure led to that patient being classified as “failure.” The effect of several variables on EDIF was investigated. These variables were: gender (male or female), age at time of surgery (three groups were defined: <40 years, 40–60 years, and >60 years), smoking habits (three groups were defined: heavy smokers, i.e., patients who smoked >15 cigarettes/day; light smokers, i.e., patients who smoked 1–15 cigarettes/day; and non-smokers), history of chronic periodontitis (three groups were defined: generalized periodontitis, i.e. patients who experienced chronic periodontitis in more than 10 sites in the mouth; localized periodontitis, i.e. chronic periodontitis that affected less than 10 sites in the patient’s mouth; and healthy patients with no history of periodontitis), and serum levels of vitamin D. Three classes of patients were considered in the analysis of serum levels of vitamin D: severely deficient patients (serum vitamin D <10 ng/mL), patients with low levels (serum vitamin D 10–30 ng/mL), and patients with optimal levels of vitamin D (serum vitamin D >30 ng/mL). Chi-squared test was used to calculate the effect of each of these variables on implant survival and a significance level of 0.05 was established. All the computations were made using SPSS 17.0.

## Results

A total of 845 patients who had been treated with Morse taper connection implants during the period 2003–2017 presented no conditions enlisted in the exclusion criteria and were therefore enrolled in this retrospective evaluation. These patients (455 males and 430 females, with an age range of 18‒94 years, with a mean age of 57.3±14.4 years at surgery, median = 58 years, 95% CI: 56.3–58.2) received a total of 1,740 Morse taper connection implants (210 patients had multiple indications for implant therapy).

The patient distribution by different groups, the related number of early failures, and the early failure rates are summarized in Table 1. Overall, there were 35 EDIFs (24 of them were attributed to lack of osseointegration and 11 to peri-implantitis). The global EDIF rate was therefore 3.9%.

**Table 1:** Number of patients, early failures, failure rates within groups, and differences among groups (chi-squared test)

	**Patients n°**	**Early failures**	**Failure rate (%)**	P-value*
**Overall**	885	35	3.9%	–
**Gender**
**Males**	455	18	3.9%	0.998
**Females**	430	17	3.9%	
**Age at surgery**
<40 years	100	5	5.0%	0.832
40‒60 years	412	15	3.6%	
>60 years	373	15	4.0%	
**Smoking habit**
Heavy smokers (>15 cigarettes/day)	98	6	6.1%	0.473
Light smokers (1‒15 cigarettes/day)	184	8	4.3%	
Non smokers	603	21	3.4%	
**History of periodontal disease**
Generalized periodontitis	97	6	6.1%	0.386
Localized periodontitis	218	10	4.5%	
No periodontitis	570	19	3.3%	
**Vitamin D serum levels**
<10 ng/mL	27	3	11.1%	0.105
10‒30 ng/mL	448	20	4.4%	
>30 ng/mL	410	12	2.9%	

*Chi-squared test, with statistically significant difference set at P<0.05.

Considering the different groups, the same incidence of EDIF (3.9%) was found in males and females, with no statistically significant differences between genders (P=0.998).

Similarly, no difference was found in the failure rate for age at surgery (P=0.832); in fact, the EDIF rates were 5.0%, 3.6%, and 4.0% for patients <40, 40–60 and >60 years of age, respectively.

Among the 282 smoking patients, 14 EDIFs were reported, for a failure rate of 4.9%. This rate was higher than that of non-smokers, the latter having only 21 early failures in 603 patients (a failure rate of 3.4%). Heavy smokers (>15 cigarettes/day) had a higher failure rate (6.1%) than light smokers (1–15 cigarettes/day; failure rate of 4.3%). However, no statistically significant difference was found in the early failure rate based on smoking habit (P=0.473).

Similarly, the incidence of early failures in patients who had a history of periodontal disease was higher than that in patients who had never been affected by periodontitis; in fact, patients who had chronic periodontitis had an early failure rate of 5.0% vs. an early failure rate of 3.3% for non-periodontitis patients. Moreover, when dividing the periodontitis patients into two categories (patients who had generalized chronic periodontitis, and patients who suffered from localized chronic periodontitis), an even higher difference emerged, with patients who had a history of generalized periodontitis displaying a slightly higher incidence of EDIFs (6.1%) than those who had localized chronic periodontitis (4.5%). However, no statistically significant difference was found in the EDIF rate with regard to previous periodontal conditions (P=0.386).

Finally, the average vitamin D serum level in the overall population was 29.5 ng/mL (±12.1, median=27, range=5–73, 95% CI: 28.7–30.2). Conversely, in patients with early implant failure, the mean vitamin D serum level was 25.4 ng/mL (±12.6, median=24, range=8–55, 95% CI: 21.2–29.5).

With regard to the different groups and the distribution of failures, a rather low incidence of failures (2.9%) was reported in patients with a satisfactorily high level of vitamin D in the blood (>30 ng/mL). The incidence of EDIFs almost doubled (4.4%) in the group of patients with insufficient serum levels of vitamin D (10–30 ng/mL) and was almost four times higher in patients with serious vitamin D deficiency in the blood (<10 ng/mL), who had a early failure rate of 11.1%. Although this represents a dramatic tendency and a clear trend towards an increased incidence of EDIF, related to the lowering of vitamin D levels in the blood, chi-squared test revealed no statistically significant difference (P=0.105) among the groups. Similar findings (P=0.160) were obtained comparing the incidence of failure in all of the 475 vitamin D-deficient patients (27 severely deficient plus 448 moderately deficient patients) versus the incidence of failure in the 410 patients with normal serum levels of vitamin D. Again, no statistically significant difference (P=0.071) was found when comparing the incidence of failure in the 27 severely deficient patients and the incidence of failure in all the other 858 patients enrolled in the study.

## Discussion

To date, only a few studies in the dental literature have investigated the correlation between serum vitamin D levels and osseointegration; most of these are experimental animal studies^37-46^ and only a few of them are clinical research in humans.^45-48^

In 2008, Alvim-Pereira et al^45^ clinically investigated the association between vitamin D receptor gene polymorphism and dental implant loss, but no relation was found.

In 2014, Bryce et al^49^ studied the relation between vitamin D deficiency and immediate dental implant placement. In this case report, it was revealed that the patient was severely vitamin D deficient and that this might have contributed to the implant failure.^49^ Two years later, Schulze-Spate et al,^46^ in a randomized, double blind, controlled clinical trial, investigated the association between vitamin D supplementation and local bone formation after maxillary sinus augmentation. They histologically compared bone samples from a group of patients who took vitamin D3 (5,000 IU) and calcium (600 mg) to another group of patients who received only calcium, six to eight months after surgery. Nevertheless, no statistically significant difference at ahistological level was demonstrated.^46^

Regardless of the positive results of vitamin D for osseointegration in some preclinical studies,^37-46^ it is still not clear whether vitamin D supplementation can effectively promote healing of the peri-implant bone.

According to Javed et al,^35^ vitamin D supplementation could stimulate bone formation and, as a result, augment the contact between the surface of the titanium implants and bone. Studies in rats proved that vitamin D deficiency could compromise the osseointegration of implants and that vitamin D supplementation stimulated bone formation.^39-42^ Moreover, the bone–implant contact ratio, bone volume around implant, and resistance of the implant to removal were improved at 2 weeks in rats suffering from kidney disease.^42^ Recent studies in animals have proved that coating the surface of the implant with vitamin D enhanced osseointegration of the dental implant.^43,44^ Nevertheless, despite the promising overtones, more studies are needed to demonstrate the efficacy of vitamin D supplementation in promoting bone healing around dental implants.

Until a few years ago, the guidelines estimated that the daily intake of vitamin D required to maintain adequate levels in the blood was 200 IU (5 mcg) in adults 19‒50 years of age, 400 IU (10 mcg) in adults 51‒69, and at least 600 IU (15 mcg) in those >70.^31,34^ These guidelines have now been revised and it is currently believed that the amount of vitamin D that should be taken daily is 2,000 IU (50 mcg) or, in certain cases (e.g., for pregnant women), up to 4,000 IU (100 mcg).^31,34^

Recently, a systematic review of the literature aimed to explore the relationship between serum vitamin D levels and periodontal disease.^50^ This review identified 365 studies and analyzed 24 of them.^50^ Seven studies fulfilled the inclusion criteria; among them, four showed the effect of vitamin D and its metabolites on periodontal health or disease.^50^ One interventional study suggested the proposed antiinflammatory role of vitamin D. Two cross-sectional studies failed to show a relationship between periodontal disease and vitamin D deficiency.^50^ The authors therefore concluded that, although some data support a protective role for vitamin D in healing of periodontium and bone, the effect of serum vitamin D levels on periodontal status remains controversial.^50^

In 2016, we published the first clinical study that retrospectively evaluated the association between low serum vitamin D and EDIF in a large number (822) of patients.^48^ In that study, we found 9 early failures (9/394 patients, 2.2%) in subjects with serum levels of vitamin D >30 ng/mL, 16 early failures (16/406 patients, 3.9%) in subjects with levels between 10 and 30 ng/mL, and 2 early failures (2/22 patients, 9.0%) in subjects with levels <10 ng/mL.^48^ Accordingly, even though we failed to demonstrate a statistically significant connection between low serum levels of vitamin D and EDIF, we did find a dramatic increase in early failures associated with low vitamin D levels in the blood.^48^ The design of that study had several advantages. First, it restricted the analysis to “early implant failures” (failures in the first healing period, i.e., within 4 months after the submerged placement of dental implants). For this reason, confounding factors such as the prosthesis and the prosthetic load, which can affect the survival and success of a dental implant, were automatically excluded.^48^ Moreover, in that study, only one implant brand (with the same thread design and the same macro- and micro-surface topography) was used, installed by the same expert operator, under the same surgical protocol.^48^ It is known that implant survival and the success of osseointegration depend on several factors: from the surgical‒prosthetic protocols,^4-8^ to the experience and skill of the operator,^5^ to the materials used (implant design and surface),^9,10,12,13^ and, obviously, to the patient’s condition.^5,6,11^ By eliminating the confounding factors related to the surgical protocols, the operator and the materials, it is possible to focus more attention on the influence of the patient’s condition, which is a key factor.^6^ The analysis of the patient’s condition is influenced by local (specifically related to the site receiving the implant, such as bone quality and quantity) and systemic factors (i.e., systemic diseases that may contraindicate the placement of dental implants).^5,6,11^ In the aforementioned study,^48^ therefore, the only confounding factor could be the local one, namely different bone quality/quantity in different patients. However, in order to limit this bias, in the inclusion criteria we excluded all the patients who had undergone regenerative bone surgery procedures before implant placement (to circumscribe the field to native bone).^48^ Moreover, patients with systemic pathologies (such as uncontrolled diabetes mellitus, immunodeficient states or bleeding disorders) or those who underwent radiotherapy and chemotherapy treatments were excluded from that study.^48^

The present retrospective study on 885 patients represents the development of the aforementioned study published in 2016^48^ and seems to confirm the evidence that previously emerged. In fact, a rather low incidence of failure (12/410 patients, 2.9%) was reported in patients with satisfactorily high serum levels of vitamin D (>30 ng/mL). The incidence of early failures almost doubled (20/448 patients, 4.4%) in the group of patients with insufficient serum levels of vitamin D (10–30 ng/mL), and was almost four times higher in patients with serious vitamin D deficiency in the blood (<10 ng/mL), who had an early failure rate of 11.1% (3/27 patients). Once again, the study showed a tendency for EDIFs to increase in patients with vitamin D–deficient states in the blood, although the difference between the three groups (P=0.105) was not statistically significant. This result is particularly interesting and, by itself, could suggest that the operator should prescribe patients an oral vitamin D supplement in the weeks leading up to the intervention, in order to increase serum values of vitamin D. This is also valid considering that, in patients in whom early implant failure occurred, the mean vitamin D serum level was 25.4 ng/mL (±12.6; median=24; range=8‒55; 95% CI: 21.2‒29.5) versus a mean vitamin D level of 29.5 ng/mL in the overall population of enrolled patients. These levels are lower than the ideal ones, and in this sense supplementation might be indicated to restore adequate levels of vitamin D in the blood.

Obviously, our present study has some limitations. First, the design is retrospective, and, as is well known, this represents an intrinsic limitation because the best design for drawing more conclusions from a scientific work is certainly the prospective one. Unfortunately, no prospective studies—nor any randomized controlled trials—are available on this topic.^47,48^ In the present study, the number of patients having severe deficiency of vitamin D (<10 ng/mL) is rather low, to the point that the presence of only one more failure in this category would have constituted a statistically significant difference among the three groups. Moreover, this study did not investigate the effects of bone quality and, if subjects with low levels of vitamin D were also likely to receive more than one implant, their risk of being classified as “failures” might have increased. And finally, the statistical analysis employed here is rather simple; a more detailed statistical analysis would be more appropriate, not only to replicate our results, but also to investigate the correlations between variables and the effects on the final outcome.

In conclusion, more clinical trials with a prospective design and appropriate statistical analysis are needed, to confirm whether or not a relation between low serum levels of vitamin D and an increased rate of EDIF exists. It would also be interesting to investigate in subsequent scientific studies whether vitamin D supplementation in the weeks leading up to surgery can reduce the incidence of EDIFs.

## Conclusion

The relationship between serum levels of vitamin D and osseointegration of dental implants has been approached in very few studies, most of them in animals. These studies have suggested that adequate serum levels of vitamin D can enhance the healing of peri-implant bone tissue. Our present retrospective clinical study aimed to investigate whether there is a correlation between low serum levels of vitamin D and EDIF. In 885 patients who had been treated with 1,740 fixtures, 35 EDIF (3.9%) were reported. No statistically significant correlation was found between EDIF and patients' gender (P=0.998), age (P=0.832), smoking habit (P=0.473), history of periodontal disease (P=0.386), and/or vitamin D serum levels (P=0.105). However, a clear trend toward an increased incidence of EDIF with lowering of serum vitamin D levels was reported, with three EDIFs (11.1%) in the 27 patients with serum levels of vitamin D<10 ng/mL, 20 EDIFs (4.4%) in the 448 patients with levels between 10 and 30 ng/mL, and 12 EDIFs (2.9%) in the 410 patients with levels >30 ng/mL. In cases where a correlation between low serum levels of vitamin D and increased risk of EDIF is proved, the clinician could administer a dose of vitamin D in the weeks before surgery. In this way, the clinician could regulate the serum levels and help increase the healing process. Obviously, prospective clinical studies and randomized controlled trials are needed to demonstrate a significant correlation between low serum levels of vitamin D and increased risk of EDIF, and thus prove that vitamin D supplementation can promote the osseointegration of dental implants.

## Acknowledgments

Not reported.

## Conflict of Interests

The authors declare that they have no competing interest with regards to authorship and/or publication of this work.

## Authors’ contributions

All authors made substantial contributions to the present study. Francesco Guido Mangano, Natale Mangano and Carlo Mangano contributed to conception and design, acquisition of data, analysis and interpretation of data; they were, moreover, involved in writing and editing the manuscript. Together, Francesco Mangano and Ana Paz were the major contributors in preparing and writing the manuscript. Sina Ghertasi Oskouei and Carlo Mangano edited and revised the manuscript before submission. All authors read and approved the final manuscript.

## Funding

The present study is self-funded.

## Ethics approval

This study was approved by the Ethics Committee of the University of Insubria (2013), with protocol number 0034086.
